# Data processing costs for three posture assessment methods

**DOI:** 10.1186/1471-2288-13-124

**Published:** 2013-10-12

**Authors:** Catherine Trask, Svend Erik Mathiassen, Jennie Jackson, Jens Wahlström

**Affiliations:** 1 Centre for Musculoskeletal Research, Department of Occupational and Public Health Sciences, University of Gävle, SE - 801 76 Gävle, Sweden; 2 Centre for Health and Safety in Agriculture, College of Medicine, University of Saskatchewan, 103 Hospital Drive, Saskatoon, SK S7N 0W8, Canada; 3 Department of Public Health & Clinical Medicine, Occupational and Environmental Medicine, Umeå University, SE-901 85 Umeå, Sweden

**Keywords:** Cost-efficiency, Exposure, Shoulder, Back, Inclinometry, Observation, Questionnaire, Work related musculoskeletal disorders, Methods development

## Abstract

**Background:**

Data processing contributes a non-trivial proportion to total research costs, but documentation of these costs is rare. This paper employed *a priori* cost tracking for three posture assessment methods (self-report, observation of video, and inclinometry), developed a model describing the fixed and variable cost components, and simulated additional study scenarios to demonstrate the utility of the model.

**Methods:**

Trunk and shoulder postures of aircraft baggage handlers were assessed for 80 working days using all three methods. A model was developed to estimate data processing phase costs, including fixed and variable components related to study planning and administration, custom software development, training of analysts, and processing time.

**Results:**

Observation of video was the most costly data processing method with total cost of **€** 30,630, and was 1.2-fold more costly than inclinometry (€ 26,255), and 2.5-fold more costly than self-reported data (€ 12,491). Simulated scenarios showed altering design strategy could substantially impact processing costs. This was shown for both fixed parameters, such as software development and training costs, and variable parameters, such as the number of work-shift files processed, as well as the sampling frequency for video observation. When data collection and data processing costs were combined, the cost difference between video and inclinometer methods was reduced to 7%; simulated data showed this difference could be diminished and, even, reversed at larger study sample sizes. Self-report remained substantially less costly under all design strategies, but produced alternate exposure metrics.

**Conclusions:**

These findings build on the previously published *data collection* phase cost model by reporting costs for post-collection *data processing* of the same data set. Together, these models permit empirically based study planning and identification of cost-efficient study designs.

## Background

Outside the research questions, goals for applicability, and knowledge translation, nothing is more central to the selection of a sampling strategy and assessment method than the cost. Despite a general consensus that the costs of data collection and processing are critical, most cost comparisons are anecdotal or qualitative [1,2], with a systematic review of the literature demonstrating lack of quantitative cost data [3]. The paucity of empirical and theoretical information on cost and cost-efficiency in research limits the decision-making power available to researchers who are planning studies.

To date, the most comprehensive review of cost-efficiency in exposure assessment uncovered only 9 articles explicitly investigating this issue, and identified several gaps to be addressed, including the simplicity of existing cost models and the lack of empirical cost data acknowledging different sources and types of cost (i.e. not every measurement has an equal cost) [3]. Since the publication of the Rezagholi and Mathiassen review, there have been contributions theorizing the relative size and importance of different cost components and the impact they may have with different exposure assessment strategies [4-7]. Quantifying the costs involved in performing different stages of research is a pre-requisite for cost-efficiency research; cost modeling, if without any concerns as to the statistical performance of collected data, has been an issue in the medical literature, for instance on cost associated with analyzing toxicology specimens in the laboratory [8] and on the benefits of computer-assisted self-interviews [9]. However, despite a few recent exceptions [10,11], empirical data, based on thorough examination of possible cost components, on the actual cost of conducting exposure assessments in occupational epidemiology is limited. These recent examinations of data collection costs associated with workplace postural exposure assessment join limited reports citing data collection costs for biomechanical exposure assessment [12].

The total cost of a study comprises many steps which we have grouped into three phases: data collection, data processing and ‘knowledge translation’ or reporting. *Data collection* may present the most obvious contribution to study cost given the cost of equipment and labour required to collect data, but this stage can be considered complete once field/lab work has ended and *raw* paper or electronic files are stored at the institution in the hands of the research team, for instance in the form of angular inclination data files obtained by inclinometer, recordings of work on video tapes, or paper copies of completed questionnaires. The *data processing* phase includes all processing of the raw data that have been collected, ending with the creation of a summary exposure database in which exposure estimates are summarized and ready for statistical analysis. Thus, in our terminology, the *data processing* phase ends before statistical analysis and reporting of results (i.e. conference presentations or drafting manuscripts). While the costs of *data collection* may be substantial [10], the data processing stage is certainly not trivial in terms of resource consumption. For example, even when ‘inherited’ files from an earlier data collection are repurposed to answer a new research question, there are still substantial amounts of labour and resources needed to produce estimates of the target exposure variables in a format appropriate for statistical analysis and hypothesis testing. The data processing phase allows for many possible strategies which can lead to increased or decreased study costs.

This manuscript complements the previously published cost model for the *data collection* aspect of exposure assessment [10] by presenting the post-collection *data processing* costs for the same data set. Given the necessity for data processing to produce exposure estimates, the availability of models for assessing the associated costs will facilitate more informed decision making on the part of researchers. For example, in our first paper, inclinometer data was shown to be the most costly posture assessment method during the data collection phase when compared to video observation and self-reported postural data. However, this rank order could change if the time and resources required for data processing are also accounted for. Both anecdotal and empirical evidence suggests that observing posture from video recordings can be both lengthy and expensive depending on the specific training requirements and observation strategies selected [7]. Thus, a cost model is required that also includes data processing costs and allows for different sampling strategy choices to be evaluated in terms of total study cost. Developing cost models expressing total cost as a function of study parameters will link previous studies investigating statistical efficiency of sampling strategies [13-15] to eventual investigations into cost-efficiency.

The overarching goal of this research is to elucidate optimal study parameters for cost efficient assessment of posture exposures. To that end, the aim of the present paper was to: i) quantify the data processing costs for shoulder and trunk posture assessment using three common methods for collecting data on working postures in ergonomics research and practice: workers’ self-reports via questionnaire, observation of video recordings of work, and direct measurement using inclinometers; ii) compare these data processing costs; and iii) develop a general cost model for data processing that can be used as a decision-making tool for planning future studies.

### Study population

The costs of post-collection data processing were determined for postural data collected over a three month period in 2011. From the pool of full and part-time employees who were not on modified duties, employees were randomly invited to participate in the study. In brief, trunk and shoulder postures were assessed for 27 airport baggage handlers on each of three days using three methods: self-report via questionnaire, observation of work recorded on video, and direct measurement using inclinometers. Three measurements were successfully collected from all but one worker who could not complete a third due to injury, resulting in 80 collected measurements. All participants gave informed consent and all methods were approved by the Regional Ethical Review Board at Uppsala University. The data collection methods are described in detail in Trask et al. [10].

## Methods

### Data collection

Data-logging triaxial accelerometers (2 M Engineering, Veldhoven, The Netherlands) were used as inclinometers and affixed to the upper back and upper arms to collect posture data for the full work shift. Questionnaires were administered pre- and post-shift to assess musculoskeletal symptoms, fatigue, and perceived postures, tasks, and workload during the work shift. Video recordings were made for either the first or second half of the shift (roughly 4 hours).

### Modeling costs

All *data collection* phase costs, such as equipment and labour associated with planning a research study, data collector training, piloting, subject recruitment, travel, and measurement at the worksite are summarized in a previously reported cost model [10]. The cost analysis presented here focuses on the post-collection *data processing* stage of research, as defined in the introduction. Thus, the cost model for the data processing phase included: acquiring or developing software to process data (in this case, video and inclinometer data), planning data processing methods, processing electronic data files (such as inclinometer files), training research assistants to process data (such as observers estimating postures from video recordings), processing data (such as viewing videos and recording observed postures), entering data for paper files (such as questionnaires), cleaning data, and summarizing each data source into exposure estimates which characterized each measurement day. Analysis costs such as those associated with performing the statistical comparisons between methods, and costs for presenting/publishing results were not included. This is strategic, since many multi-centre or multi-investigator studies use the ‘complete, cleaned database’ as a milestone for commencing the reporting phase of a study.

The general model for assessing the total data processing cost for method *m*, C_P*m*_, included both fixed costs (denoted by a hatch: Č):?

• the cost of study administration and planning, including meetings, documentation of methods development, and correspondence (Č_A_);

• the cost of developing and/or refining (custom) software (Č_S_);

and variable costs (denoted by a dot: Ċ):

• the cost of training data processors for method *m* (Ċ_T_);

• the cost of actually dealing with (managing) the collected data (Ċ_M_).

This model can be applied to any measurement method, *m,* following the general Eq. (1):

(1) $$C_{Pm}={Č}_{A}\;+\;{Č}_{S}\;+\;{\dot{C}}_{T}\;+\;{\dot{C}}_{M}$$

The cost of training observers to estimate postures from video films, Ċ_T_, was calculated as the product of the number of trainees (n_T_) and the unit cost per trainee (ċ_T_), summed with the product of the number of instructors (n_I_) and the unit cost per instructor (ċ_I_):

(2) $${\dot{C}}_{T}=n_{T}\;{\dot{c}}_{T}\;+\;n_{I}\;{\dot{c}}_{I}$$

Although the length of training time is not explicit in this equation, the costs ċ_T_ and ċ_I_ depend on the length of training and so can be considered specific to a particular study. The development of training curriculum was considered as a fixed cost included in the costs of planning, Č_A_.

The cost of actually dealing with data, Ċ_M_, was calculated as the product of number of worker-day files, n_F_, and the unit cost of handling each file, ċ_F_.

(3) $${\dot{C}}_{M}=n_{F}\;{\dot{c}}_{F}$$

As with the length of training, the value of ċ_F_ is specific to a particular study because it varies with the amount of working time aggregated into an exposure summary variable. Alternative ways of treating data can produce different costs per file. As a special example of a method for which the unit cost per file can be altered based on sampling strategy, the video data managing cost, Ċ_M_, depends on the resources invested in observing each video. To account for variation in handling costs due to the video sampling strategy selected, the term Ċ_M_ can be further detailed in terms of additional observation sampling parameters, as shown in Eq (4):

(4) $${\dot{C}}_{M\mathrm{observation}}=n_{F}\;n_{R}\;n_{O}\;{\dot{c}}_{V}$$

Where n_F_ is the number of video files, n_R_ is the average proportion of a video reviewed by each observer, n_O_ is the number of observers, and ċ_V_ is the unit cost for analyzing one complete video file once. The cost ċ_V_ is specific to a particular video observation study and depends on both the number of hours of video collected and the sampling rate (for a work-sampling approach) or segment length (for event registration or exposure-averaging approach) for analysis [11]. The proportion of video reviewed, n_R_, is defined as the average proportion of still frames or video segments from any video file in the complete data set that an observer actually reviews. If, for instance, an observer reviews half of the collected videos or half of all the video frames collected, n_R_ will be 0.5. Thus, the “coverage” product n_R_ n_O_ measures the total observation effort devoted to each video file, with 1.0 corresponding to one observer observing all frames or segments once (or, similarly, two observers each reviewing half the frames). If each video is reviewed completely by two observers, n_R_ n_O_ will be 2.0. Thus, the product n_R_ n_O_ reflects that observers may make repeated observation of the same video frames, which is one way of improving precision of exposure estimations obtained by observation [7].

Substituting Eq.s (2) and (4) into Eq. (1) results in models specific to the observation method:

(5) $$\begin{array}{l} C_{P\mathrm{observation}}={Č}_{A}+{Č}_{S}+n_{T}\;{\dot{c}}_{T}+n_{I}\;{\dot{c}}_{I}\\ \;+n_{F}\;n_{R}\;n_{O}\;{\dot{c}}_{V} \end{array}$$

Further, substituting Eq.s (2) and (3) into Eq. (1) results in models specific to the inclinometer and questionnaire assessment methods:

(6) $$\begin{array}{l} C_{P\mathrm{inclinometer}}\;\mathrm{and}\;C_{P\mathrm{questionnaire}}={Č}_{A}+{Č}_{S}+n_{T}\;{\dot{c}}_{T}\\ \;+n_{I}\;{\dot{c}}_{I}+n_{F}\;{\dot{c}}_{F} \end{array}$$

### Collection of cost data

Prospective time tracking was used to collect data processing cost data, as described in Trask et al. [10]. Briefly, researchers tracked the amount of time spent performing office and lab tasks related to data processing (such as meetings, administration, and software development) using a custom Excel macro with pre-defined task categories. For each research staff member working on the project, entries were later compiled into total hours spent per task category. Processing time was tracked separately for each method. Video analysis by the observers rating the video films was tracked using an internal time counter in the analysis software, which gave the analysis time for each frame: from this data an average time per frame was calculated. All costs were standardized to Euro currency using the average exchange rate between March 2011 through October 2012. Researcher time was valued at €31 per hour, video analyst time at €23 per hour and data input staff at €20 per hour. All labour costs required an additional, university-specific overhead of 68% bringing the total labour and overhead costs to €52, €39, and €34 for researchers, video analysts, and data enterers, respectively. All the costs considered in this analysis were borne by research grants.

### Inclinometer data processing

During the initial data collection phase, inclinometer posture information was collected over the full duration of each shift at 32 Hz. Of the 81 planned measurements (27 workers × 3 days), 80 were successfully collected (loss of one due to worker drop-out), and 79 were successfully processed (loss of one due to intractable noise). Full-shift recordings were exported from the VitaMove data collection software, downsampled to 20 Hz, and the file format was converted to permit data analysis using software developed at the Department of Occupational and Environmental Medicine, Lund University, Sweden [16,17].

**Figure 1:**
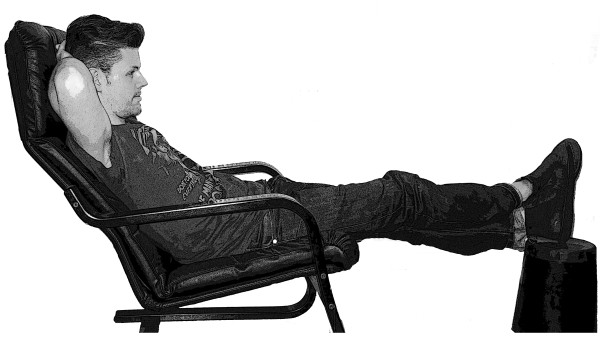
**Example of trunk and upper arm postures that were deemed necessary to correct.** Permission to publish this image has been secured from the individual.

Tri-axial accelerometer inclinometers measure sensor orientation with respect to the line of gravity. As such, reclining trunk postures (Figure 1) would be recorded as extreme extension. To address this nuisance, an algorithm was developed to identify such reclined postures and replace the extreme angles measured by the inclinometers with 0˚ (trunk) and 10˚ (shoulder).

A custom Excel Macro was developed to summarize shift-long data into exposure metrics, such as: mean, standard deviation, selected values from the cumulative amplitude probability distribution function (APDF) of the trunk and arm inclination angles, the frequency per minute of excursions over a certain threshold (i.e., 45 degrees), and the frequency per minute of excursions over a certain threshold that exceed a certain duration (i.e., 45 degree inclinations lasting longer than 5 seconds). Combinations of posture and velocity were used to assign characteristics such as ‘resting’, ‘extreme’, ‘static’ etc. following the definitions used in previous inclinometer studies [18,19].

All raw file processing and summary variable calculations were conducted by a single researcher who was also involved with the team performing the software development and refinement, meaning that for the inclinometer data, there was no additional training time required. The inclinometer data were considered complete when work shift summaries had been compiled for all 79 usable measurement-days.

### Observation data processing

During the initial data collection phase, half-shift video recordings (4 or more hours) were made for each worker equipped with inclinometers during that shift. To process this data, four individuals were recruited from the student population at the University and were trained to be video observers. The observers had varying experience in ergonomic exposure assessment, but all were given standardized training in posture evaluation, exposure category definitions, interpretation of video images and use of the software. Observers first evaluated still frames as a group facilitated by one of the researchers to ensure consistent application of definitions. Observers then performed approximately 16 hours of practice with the method, supervised by the researcher who trained them, which included time and opportunity to ask questions. Group discussions were used to harmonize interpretations of difficult or unusual images.

**Figure 2:**
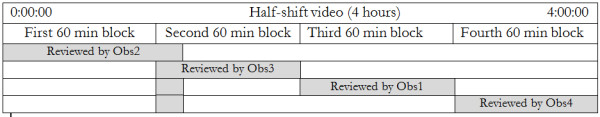
Example of the allocation of the shift between four observers, including the ‘overlap’ section to assess inter-observer agreement.

Videos lasting longer than 4 hours were truncated to 4 hours; videos shorter than 4 hours were reviewed completely. The observers subsequently analyzed all 80 half-day (4 hour) video recordings to categorize trunk and shoulder postures. Each of the four observers analyzed a 60-minute portion of each of the 80 work shifts; video recordings were assigned using a randomized order of worker-days and assigned shift portions. To investigate the inter-observer agreement and, at the same time, improve the precision of the eventual exposure estimates, one 15-minute portion of each half-shift recording was analyzed by all four observers; the overlap block always occurred during the first 15-minute portion of the second block of video and resulted in 3 of the observers rating an additional 15-minute block. An example of the observation of a work shift is shown in Figure 2.

**Figure 3:**
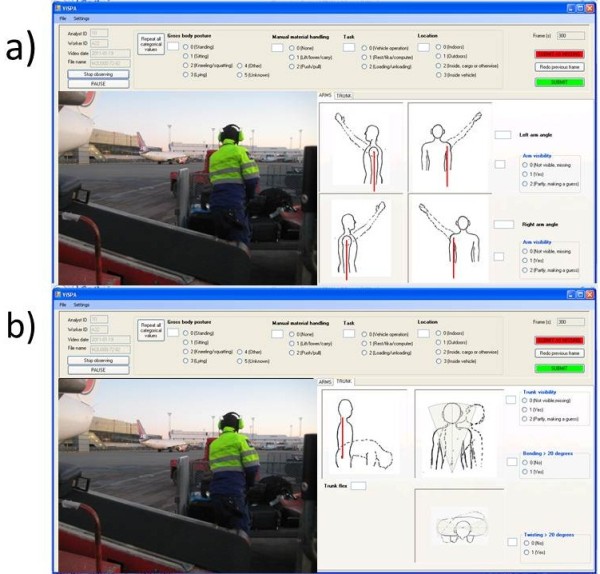
Views of the ViSPA observation software operation for the shoulder (a) and trunk (b).

A work sampling observation approach within each 60 minute block whereby a custom software program presented still images to observers selected according to a 55-second interval approach. To account for the equipment setup time which occurred in block 1, the video observation on the first block started 300 seconds into this block. Observers coded all still images for the following categorical variables: gross body posture (standing, sitting, kneeling/squatting, lying, and other.); work task (vehicle operation, loading/unloading, computer work/breaks, other), presence of materials handling (lift/lower/carry, push/pull); location (inside vehicle, inside plane, indoors, outdoors); and the presence of trunk twisting or lateral bending greater than 20 degrees (yes/no). Observers coded at a self-selected pace using either a mouse point-and-select or a key-and-tab input method. Left arm, right arm, and trunk postures were recorded as continuous variables (-180 to +180 degrees) by dragging a ‘posture line’ on a mannequin, similar to the method described by Bao et al. [20] (see Figure 3). Trunk flexion/extension angle was assessed with respect to gravity. Shoulder posture was assessed in terms of inclination with respect to gravity. The degree of visibility for each body part was also evaluated categorically by the observers (clear, completely undistinguishable, or inferred - i.e. arm in line with the body but obscured by the body). The software automatically recorded the amount of time required by an observer to analyze each frame.

Observational data were submitted to the same replacement procedure as the inclinometer data to exchange “extreme” (with respect to gravity) trunk extension and arm elevation postures occurring during rest with neutral values to better represent the exposure at these times.

The fixed-interval sampling strategy with 55s between frames yielded a maximum of 261 unique frames per half-shift of video ({4 h × 60 min × 60 s}/55s), and 783 frames per worker for all 3 half-shifts. Excluding the re-watched portion of the files, this amounted to 20,880 frame observations (261 frames × 80 collected worker-days). These observations were then summarized into work shift exposure variables similar to those generated from the inclinometer data: mean, standard deviation, APDF values for trunk and arm angle, the number of excursions over a certain threshold (i.e., 45 degrees), and the proportion of time spent in a certain posture (i.e. laterally bent), as well as cumulative time for only observed categorical variables such as location (i.e. in the cargo hold of a plane). Some variables generated from the inclinometer data, such as movement speed, were not possible to obtain from the observed posture data because of the nature of the still frame method. Observation data were considered fully processed when the cleaned database including 80 work-shift summaries was complete.

### Questionnaire data processing

During the initial data collection phase, workers filled out pre- and post-shift questionnaires to report musculoskeletal symptoms and fatigue levels. The post-shift questionnaire also asked workers to report information on the duration of tasks performed and postures assumed during their shift, as well as what assistive equipment, such as conveyor belts and baggage wagons, they had used during their shift. The post-shift questionnaire was specific to the workers’ department (ramp versus sorting hall) to allow for recognizable, job-specific vocabulary and tasks. In the case of ramp workers, an additional flight diary was filled out detailing the type of flight, total amount of baggage and cargo for each flight, the worker’s role in loading that flight, and the use of any assistive equipment. A total of 230 questionnaires were collected, amounting to a total of 696 pages (54 × 8-page diary + 88 × 3-page pre- and post-shift questionnaire). Data from the paper copies were entered by skilled, trained research administrators and double-checked by researchers. Questionnaire data were considered fully processed after data cleaning was performed on this completed database.

### Simulations based on study costs

In addition to evaluating the three specific data processing procedures employed in the current, original study, the cost models presented (Eq.s 5 and 6) can be used to simulate the costs of different alternative measurement strategies, and thus can be used as a decision tool. To demonstrate the effect of altering different design parameters on the processing cost for a given method, six alternate study scenarios were investigated, including three cases where observation-specific design parameters were manipulated (designs 4-6) and one case where inclinometer and questionnaire design parameters were manipulated (design 7), and the sensitivity of the total cost to these renewed inputs considered. The parameters for these scenarios are listed in Table 1. The study scenarios examined were:

1) The current study

2) The case where the methods are well-established to the research group, so no software development, acquisition or learning is required, and fixed costs were limited to planning and administration costs.

3) The case where double the number of work-days were collected

4) The case where all 4 observers watch the entirety of each video file (fully replicated observation across observers)

5) The case where only 2 hours of video per work shift are analyzed and there is no duplication within or between observers

6) The case where ċ_V_ (and therefore ċ_F_) doubles, for instance because the time to analyze each video frame or segment doubles, or because the number of frames to be observed per video doubles.

7) The case where quality control and examination of the raw data is decreased such that the ċ_F_ for inclinometer is half of current values. Similarly, the case where the questionnaire is half as long, requiring half the data entry and therefore entailing half the current value of ċ_F_ for questionnaire.

**Table 1:** Study parameters for alternative study scenarios simulated with the cost model

**Scenario**	**n**_**T **_**number of analysts trained**^**a**^	**n**_**I **_**number of instructors**^**a**^	**n**_**F **_**number of work shift files**	**n**_**O **_**number of observers**^**b**^	**n**_**R **_**average proportion of 240 min video reviewed per observer**^**b**^	**ċ**_**F **_**unit cost of processing one file (multiples of current study)**
Design 1: current study	4^b^/2^a, c^	2^b^/1^c^	80	4	0.30	1
Design 2: no training or software costs	**0**	**0**	80	4	0.30	1
Design 3: double the work shift files	4^b^/2^a, c^	2^b^/1^c^	**160**	4	0.30	1
Design 4: full observation overlap	4^b^/2^a, c^	2^b^/1^c^	80	4	**1.0**	1
Design 5: 2 hours observed per half-shift	4^b^/2^a, c^	2^b^/1^c^	80	4	**0.125**	1
Design 6: longer observer analysis time^b^	**4**^**b**^	**2**^**b**^	80	4	0.30	**2**
Design 7: shorter analysis time for inclinometer recordings and questionnaires	**1 **^**a **^**/2**^**c**^	**0 **^**a **^**/1**^**c**^	80	4	0.30	**0.5**

^a^inclinometer.

^b^observation.

^c^questionnaire.

Items in bold represent parameters which are different than the present study.

### Combining data processing and data collection cost models

As mentioned above, we have suggested in a previous paper a model for calculating costs associated with the *collection* of raw data, using method *m* (Trask et al. [10]). This model is as follows:

(7) $$\begin{array}{l} C_{Cm}={Č}_{A}\;+\;{Č}_{R}\;+\;{Č}_{E}\;+\;{Č}_{T}\;+\;{\dot{C}}_{V}\\ \;+\;{\dot{C}}_{H}\;+\;{\dot{C}}_{R}\;+\;{\dot{C}}_{D} \end{array}$$

The model includes fixed costs of project meetings, administering, and planning (Č_A_), and the fixed cost of training data collectors to use measurement method *m* (Č_T_). Similar cost components are included in the model for data processing suggested above (Eq 1), even though training costs are, in this case, considered variable. C_C*m*_ (Eq. 7) also includes the fixed costs of recruitment (Č_R_); and the fixed capital cost for equipment (Č_E_), along with the variable costs of traveling to the worksite (Ċ_V_); hotel accommodations during overnight trips (Ċ_H_); the cost of recruiting workers at the worksite (Ċ_R_); and the cost of onsite data acquisition (Ċ_D_).

Combining Eq.s 1 and 7 provides a model for the total study cost, C_*m,*_ (Eq. 8) for any particular method *m*, including both data collection and data processing phases. Administration (Č_A_) and training (Ċ_T_ - Eq 1; Č_T_ - Eq.7) terms appear in both models, thus these cost components can be combined.

(8) $$\begin{array}{l} C_{m}={Č}_{A}\;+\;{Č}_{R}\;+\;{Č}_{E}\;+\;{Č}_{S}\;+\;{\dot{C}}_{T}\\ \;+\;{\dot{C}}_{V}\;+\;{\dot{C}}_{H}\;+\;{\dot{C}}_{R}\;+\;{\dot{C}}_{D}\;+\;{\dot{C}}_{M} \end{array}$$

## Results

### Study costs by data measurement method

The total data processing costs for each of the three measurement methods utilized in the current study are presented in Table 2. The first column in Table 2 (‘applicable to all methods’) shows the costs that are required no matter which method is used; the total for each method represents the total study cost for applying that method, including the costs in the first column. The data processing cost was lowest for questionnaire data, at less than half the cost of the other two methods (Table 2). Estimated data processing costs were approximately 20% larger for posture exposures obtained via observation of video data than from inclinometers.

**Table 2:** Data processing cost components for the three measurement methods in the current study

**Cost component**	**Applicable to all methods**^**a**^	**Inclinometer**	**Observation**	**Daily exposure questionnaire**
**Fixed costs**				
Č_A_ planning and administration	€ 8,245	€ 5,114	€ 8,639	€ 510
Č_S_ software	€ 0	€ 10,924	€ 3,479	€ 613
Sum of fixed costs (Č_A +_ Č_S_)	€ 8,245	€ 16,038	€ 12,118	€ 1,123
**Unit costs**				
ċ_T_ unit cost of training trainees	€ 0	€ 0	€ 920	€ 510
ċ_I_ unit cost of instructors	€ 0	€ 0	€ 1,288	€ 794
ċ_F_ unit cost of file analysis	€ 0	€ 25	€ 50	€ 16
**Variable costs**				
Ċ_T_ training costs	€ 0	€ 0	€ 6,256	€ 1,815
Ċ_M_ costs of managing (handling) data	€ 0	€ 1,972	€ 4,012	€ 1,307
Sum of variable costs (Ċ_T_ + Ċ_M_ )	€ 0	€ 1,972	€ 10,268	€ 3,122
**Totals by method**				
Total fixed and variable costs (column sum of fixed and variable costs)	€ 8,245	€ 18,010	€ 22,385	€ 4,245
C_P*m*_ total data processing cost^b^		€ 26,255	€ 30,630	€ 12,491

^a^costs that are required no matter which method is used.

^b^represents the total data processing phase cost for applying that method, including the costs in the first column.

### Costs by study scenarios

The total data processing costs estimated using the six different study scenarios are presented in Table 3. Among the simulated study scenarios, the relative rank of processing costs by measurement method was consistent; questionnaires had the lowest cost for all scenarios. Observation was consistently the most costly in terms of data processing, with costs ranging from 1.1- to 1.6-fold higher costs than inclinometry and 2.1- to 3.4-fold higher than questionnaires.

**Table 3:** **Total data processing costs, C**_**P** ***m***_**(cf. Eq. 1) for actual study and six simulated study scenarios**

**Scenario**	**Inclinometer**	**Video observation**	**Daily exposure questionnaire**
Design 1: current study	€ 26,255	€ 30,630	€ 12,491
Design 2: no training or software costs	**€ 15,331**	**€ 21,698**	**€ 10,062**
Design 3: double the work shift files	**€ 28,227**	€ 36,248	**€ 13,799**
Design 4: full observation overlap	€ 26,255	**€ 42,667**	€ 12,492
Design 5: 2 hours observed per work shift	€ 26,255	€ 28,625	€ 12,492
Design 6: double observation time of videos	€ 26,255	€ 36,248	€ 12,492
Design 7: half the processing time for inclinometer recordings and questionnaires	€ 25,269	€ 30,630	€ 11,839

Maximum and minimum modeled costs shown in bold for each measurement method.

### Combined data collection and processing costs

The total study costs for both data collection and processing phases are presented in Table 4 for all three measurement methods. Self-reported questionnaire data proved to be the least costly for each phase and thus for the combined cost. Inclinometer and observation data measurement methods proved very similar in combined cost with a higher cost of *collection* of inclinometer data and the higher cost of *processing* for the observation data. When the data collection and data processing costs were combined, the inclinometry method remained the more costly, but only slightly: the combined costs were 7% higher than the observation method. The questionnaire method’s combined costs remained lowest; inclinometry costs were 57% higher than questionnaires in the current study scenario (Table 4).

**Table 4:** Combined data collection and data processing costs from the current study

**Cost component**	**All**^**a**^	**Inclinometer**	**Observation**	**Daily exposure questionnaire**
C_C*m*_ Data collection costs (Eq. 7)*	€ 28,854	€ 36,475	€ 26,515	€ 8,011
C_P*m*_ Data processing costs (Eq. 1)	€ 8,245	€ 18,010	€ 22,385	€ 4,246
Data collection and data processing costs (sum of two first rows in the column)	€ 37,099	€ 54,485	€ 48,900	€ 12,257
C_*m*_ Total Combined Study Cost (Eq. 8)^b^		€ 91,584	€ 85,999	€ 49,357

^a^costs that are required no matter which method is used.

^b^represents the total study cost for applying that method, including the costs in the first column.

*as reported in Trask et al. [10].

## Discussion

### The impact of fixed and variable costs

Simulated study scenarios provided insight into the relative contributions of different types of costs, and can act as a sensitivity analysis of the components in the overall cost model. Altering the number of files processed or the research infrastructure available to researchers can change not only the cost of conducting a study with a given method, but also the cost of a method relative to other methods.

The fixed costs associated with developing processing methods ‘from scratch’, as in the present study, are unique to the first time a research group uses a new method. Software development and training costs had a big impact on data processing costs for both the observation and inclinometer methods, as shown by the decrease in processing cost when they were eliminated in design 2 (Table 3). Once all the protocols, software, and trained personnel are in place, any additional data can be processed at a substantially lower cost per data unit. Thus, after the initial investment, it will be possible to process data from many different worksites and sampling campaigns with lower marginal cost (marginal cost is the cost of one additional measurement). However, in the case of a method for which the training costs were high, the likelihood of this scenario decreases over time as turnover in trained research assistants may occur. The same may also occur, to some extent, for methods requiring continued software upgrades and developments in the desired summary.

As an extension of this concept, the nature of fixed and variable costs means that the marginal cost of an additional measurement can be small relative to the total cost of the study. For example, design 3 has double the number of work shift files than the current study (design 1), but the processing cost is only 7-15% higher, depending on the measurement method employed. The impact of including or excluding fixed costs has been previously demonstrated for observation method strategies [11]; depending on the research questions, over-sampling may prove most cost effective and a limited increase in cost could provide some ‘insurance’ if a power calculation used to plan the study is developed according to uncertain assumptions [21]. Design 3 doesn’t change the relative ranking of method costs either, but shows the total inclinometer processing cost-per-measurement decreases much faster than for the observation method. This is a result of the variable cost of data processing being lower for the inclinometer method (€ 25) than for observation (€ 50 in the current study scenario). Given this higher handling cost per file, ċ_F_, further increases in sample size would eventually result in a higher total combined data collection and processing cost for the observation method compared to the inclinometer method. The cost per file can be further increased or decreased based on the researcher’s tolerance for misclassification; persevering in trying to deal with a few noisy raw data files can substantially increase the average variable costs per file, i.e. the unit cost of handling one file.

Design 7 (Table 3) demonstrates the situation where the total cost of data processing inclinometer and questionnaires is less because it takes less time to process each data unit. However, adopting this processing strategy shows only modest reductions in inclinometer and questionnaire costs of about 4%. Limiting the quality assurance, visual inspection, and time-intensive data cleaning for electronic data may seem counter-intuitive among researchers trained to be meticulous. However, it is worth considering as an option in the case where cost savings can be considerable and data quality losses are likely to be low. It may be that the improvements in data quality gained from a highly time-consuming data processing are not worthwhile; however, the impact on the precision of the exposure variables would need to be considered in light of the aims of a particular study. Cutting the inclinometer data processing time in half (design 7) yielded a savings of roughly 5%. Without considering data collection costs, the processing savings could be spent on collecting additional measurements (in the present study, 80 additional measurements could be had for the same cost): such a strategy may have a larger, positive impact on exposure estimate precision than utilizing fewer, more highly-processed measurements. The impact of simpler processing strategies on cost efficiency is worthy of future investigation.

Cutting data processing time in half (design 7 in Table 3) led to only a 5% decrease in questionnaire costs compared to the current study. Less processing time could result from including fewer variables on the questionnaire, administering fewer questionnaires per worker (i.e. only post-shift), or, possibly, from questionnaire designs resulting in easier data input. Limiting the number of questions on a questionnaire will decrease the processing time, and could also affect data collection in that workers may be more willing to participate, or may be more motivated to consider each question and provide higher-quality estimates; there is likely a lower limit to the number of questions that could be considered to yield useful information. Although the effect on worker participation was not investigated in the current study, it is worth considering since questionnaire length has been shown to affect both participation rates and length of time spent on questions [22]. Savings may also result from a change in self-report format; tablet computers have been used successfully for self-administered questionnaires in a health-care context [23], and smartphones are an obvious choice for “diary” questionnaires focusing on events or experiences occurring several times every day. This technology could be used in a workplace setting as well, resulting in virtually no data entry costs. However, there could be considerable fixed costs for equipment and software, meaning that this strategy is most appropriate for large sample sizes (or multiple studies using the same method) where the high fixed costs could be spread over many measurements.

Our previous report of data collection costs predicted that efforts related to processing and analysis would likely change the relative cost ranking of posture assessment methods [10], and that is borne out in the processing results reported here. The inclinometer method was the most costly when only the data collection phase was considered, while the observation method was the most costly when only the data processing cost was considered. When both collection and processing phase costs were combined, the difference between these two measurement methods diminished to approximately 7%. However, different study scenarios can alter this considerably. For example, if data collection were to be replicated immediately after the ‘start from scratch’ situation of the present study (as described in design 5 of the previously-published cost paper [10]), there would be no fixed equipment cost. Since the fixed equipment cost for the inclinometer was higher than for observation, this situation would decrease the combined cost of inclinometer relative to the observation. In some studies, data collection costs may be negligible compared to costs for processing data. For instance, it is not uncommon for studies of occupational exposure to use previously-collected data to investigate a new research questions; for instance for studies of sampling strategies [11,13,21,24]. In this case, the costs for collecting the raw data have already been accounted for by the original study, so only the data processing costs need be considered when selecting a method.

### Observation processing: a special case

Video observation, as applied in this study, was the most costly processing method. However, video observation presents many sampling strategy options, resulting in a wide range of possible processing costs. For this reason, design scenarios 4-6 focused on changes to the observation processing strategy. Overlap (i.e. repeated views of the same frame by different observers) increases the precision of a group mean when the between-observer variance is high [7,25], and repeated observation is shown to be a cost-efficient alternative to collecting more video films of the work [7]. Increasing the “coverage” of each video film (i.e. n_R_ n_O_, cf. Eq. 6) from 0.31 to 1.0 yields a fully-balanced design, as described in design 4. This design maintains the same number of unique frames, but all four observers analyze all frames, and results in nearly a 2-fold increase in the observation processing cost when compared to the current study scenario. When the combined collection and processing costs are considered, such a complete overlap design would result in an 11% cost increase when compared to the current study scenario. It remains to be seen whether this is a cost-efficient strategy.

Decreasing the number of hours of video analyzed per work shift, as in design 5, had several effects. Such a design decreased the processing cost to 91% of the current study scenario, increased the relative contribution of the fixed costs, and decreased the amount of exposure information available from which daily summary variables were made. This design strategy might seem appealing as a pilot study where budget is more limited and looser estimates are acceptable. However, since this method delivers less than half the data for only a 9% reduction in price, it seems unlikely to be as cost-efficient an option.

Video analysis time depends on the number of exposure variables which observers must evaluate; and while it could be faster to record and analyze only a single variable, this study took a multi-variable approach and, further, asked observers to analyze postures on a continuous angle scale (i.e. not ordinal or nominal) for both upper arms and trunk flexion, in addition to the nominal variables utilized for trunk rotation, trunk lateral flexion, presence of manual handling, task, and location. The average length of time to analyze a single frame was 22 s (median = 20 s; standard deviation = 18 s), but this average includes many “missing” frames, representing cases where the worker was in the rest room, moving behind a vehicle or object, or otherwise not visible in the frame and for which observers were required to only click a single button indicating the absence of the worker. Missing frames accounted for 13.7% of all analyzed frames, and the average time for observing a missing frame was 2.1 seconds. Design 6 investigates the case where either the time interval between frames to be analyzed in each video is decreased by half, or the analysis *per se* of each frame take twice as long (i.e. about 45 seconds) which could occur if the number of variables increases, the proportion of missing frames decreases, or the complexity of frames increases. The cost increase demonstrates the impact these video analysis characteristics can have on a research budget.

### Performance of the data processing cost model

Compared to the previously-published 9-term model for data collection phase costs, the 4-term data processing phase model presented in the present study seems relatively parsimonious. All four data processing cost components included in the proposed model (Eq. 1) lend to differentiating between the relative cost efficiencies of the different measurement methods; the simplification of the model to exclude any of these components would thus give an incomplete picture of costs and limit good decision making. If there was a large increase in the number of work shifts processed, as simulated in design 3, fixed costs would start to contribute less to total study cost and the average cost per measurement would decrease. However, the non-trivial marginal costs of data collection mean there are still practical limitations to the number of measurements that can be included in a study. This can be seen in published reports on biomechanical exposure assessments using observational or technical measurements in occupational life, where samples of approximately 100 work shifts are somewhat common [19,26,27], but samples of 200 or more are rare [28].

The relative influence of researcher labour costs on the total study costs is more predominant in the *data processin*g phase than in the *data collection* phase due to the lack of equipment and travel costs associated with the processing phase. However, embedded in the *data processing* phase labour costs are several implicit equipment/infrastructure cost components, as outlined by Rezagholi et al. [11]. For example, energy consumption, computers and IT support, institutional building fees and maintenance are all included in the overhead cost term. This is a cost that the university levies on top of research labour salaries (and was a 68% increase in the present study), rather than comprising unique, explicit terms in the model. Similarly, taxes and benefits are included in the hourly wage used to calculate labour costs, so the effect of tax, workers’ health insurance, and benefits are not explicit in the model components. This modeling decision simplified cost calculations, but those working with different overhead assessment procedures or different social security systems will need to adjust the model accordingly.

The cost tracking methodology itself could affect the fixed and unit cost inputs to the model, thereby impacting the ability to accurately make predictions. To determine labour time, all researchers involved were asked to track the time they spent on tasks related to this study. This task was to be done in addition to their regular work tasks, and despite high motivation on the part of the researchers to collect the data, time reporting may have been forgotten or grossly approximated afterwards. Indeed, researchers describe re-creating some events retrospectively on their spreadsheets as they forgot to track the event as it was happening. Forgetting to track activities may have introduced bias by underestimating the time spent and thus the total cost of study. However, when double-checked against the percentage time allotted to this project for each researcher (an administrative requirement of their respective institutions), the time spent was on the order of predicted values. Research staff time for data entry was tracked as part of the institution’s accounting and payment protocols. It is anticipated that these individuals were very motivated to track and enter the whole amount as they were paid only for the time they reported. This self-reported work time process was familiar to these individuals and they were well-practiced, so a low level of reporting error is anticipated. The observation time is also anticipated to have very low error since it was tracked by the computer program and required no user input. There were very few occasions where the analysis time for a frame exceeded 5 minutes. All such events were assumed to represent occasions where observers took a break without pausing the program, as they had been instructed to do; these events were therefore removed when calculating the average time to evaluate a frame. Despite our best efforts to track labour costs, it is not possible to know with high precision the exact amount of time spent on each tasks. Nonetheless, the methods used here deliver a reasonable estimate of the labour costs associated with data processing.

### Validity: considering the value of posture assessments

It must be made clear that the current study does not evaluate the quality of data produced by the current posture assessment methods. The measurement methods compared clearly differ in the nature of information delivered: different dimensions of exposure, posture captured via continuous or categorical variables, different breadth and depth of exposure information, and different origin of information (worker vs researcher vs electronic equipment). These methods are also likely to vary in both accuracy and precision, and cost alone is not sufficient to make a decision about which posture assessment method should be used. For example, self-report remains the least costly option in terms of data collection, processing, and total cost. However, self-report is generally placed at the bottom of the exposure assessment ‘validity hierarchy’ which lists direct measurement at the top [1,2,29,30]. Although this hierarchy forms the basis of most exposure assessment method validation studies [31,32], it is rare to find validation studies that quantitatively demonstrate a difference in precision between methods rather than just agreement.

The ‘validity hierarchy’ has previously been linked to price, either explicitly or implicitly, where direct measurement is axiomatically said to be more costly than observation, which is in turn more costly than self-report methods [1]. The higher data processing cost demonstrated in this study for observation measurements compared to inclinometer measurements indicates that larger samples would favour the inclinometer method. Even with the current study scenario, the similar total study cost between these two methods indicates that the selection of inclinometer versus video observation method does not have to be made simply based on cost, but can be made based on the desired quality or nature of the exposure data produced. Furthermore, the axiom that the ‘best’ quality data – assumed in the hierarchy to be obtained by inclinometers – is also always the most expensive does not appear to hold true (at least at the processing stage), and thus this axiom must be reconsidered. It has already been suggested that self-report and observation allow for a wider scope of variables than direct measurement tools like inclinometers [1,12]. However, these methods are more subjective than direct measurement. Self-report, for example, summarizes exposure over a period of time, typically, but not necessarily, over a whole day, and is based on a retrospective report of the postures and tasks encountered during that day. In comparison, the observation method could generate over 200 observed video images during a similar period of time, and the inclinometer method, many thousands of inclinometer samples (8 hours × 3600 seconds per hour x 20 Hz sampling rate). Further, the inclinometer method provides speed of movement data that is not accessible via video frame analysis with a 55-second sample interval as in the current study, although this would be possible using other video sampling strategies based on expert assessments of speed from video segments. The volume of timeline data delivered by the inclinometer and observation methods allow more versatility for post-hoc development of research questions via alternate processing strategies such as resampling within collected data [13]; this would be harder to perform with the self-report data.

In order to optimize cost-efficiency of different methods and identify the best method for a given purpose and at a given budget, we need data on the statistical efficiency of the measurement methods in addition to cost data [6,7,11]. We propose that the cost data presented in this paper be combined with exposure variance component estimates in order to compare exposure assessment methods in terms of cost-efficiency. This strategy would permit the usage of already published literature reporting physical exposure variance components. For example, there already exist reports of biomechanical exposure variance components for diverse occupations such as hairdressers [15], office, custodial, and maintenance workers [33], and heavy industrial occupations [34]. There are also some studies using variance components to estimate precision of exposure assessments [14,15,35,36]. This new overall efficiency model option is thus immediately available to researchers, albeit there has been only limited cost data published to date, and studies that combine both cost and measurement precision will make a rich avenue for future investigation [3]. A further challenge would be to fully understand the costs and cost-efficiency of exposure assessment strategies that combine more than one method, as for instance when combining self-reported or register data on jobs and/or tasks from “many” subjects with direct measurements of exposures in these jobs and tasks on a limited representative sample of subjects [26,37-39].

## Conclusion

Observation of video was the most costly data processing method with total cost of € 30,630, and was 1.2-fold more costly than inclinometry (€ 26,255), and 2.5-fold more costly than self-reported data (€ 12,491).

The findings presented here complement the previously published cost model for *data collection* phase costs [10] by reporting the empirical costs for the post-collection *data processing* phase of the same data set. Study findings indicated that video observation had the highest processing costs (**€** 30,630), which resulted in very similar *combined* collection and processing phase costs for both the observation (€ 85,999) and inclinometer (€ 91,584) methods, despite much higher data collection costs for the inclinometer approach. Further, at large sample sizes, the data processing costs can become so large for the observation method that inclinometer data becomes more cost effective. This finding is in contrast to the notion that higher quality data is inherently more costly. Self-report was the cheapest method on all levels, however it produces very different output exposure variables than the other methods, and thus, it may be difficult to compare the informative value of the data obtained from the three methods. Together, the presented cost models allow for better study planning by identifying the fixed and variable costs of each measurement method, and the effect on the total cost of collecting more or fewer measurements. The cost models and the underlying study data are critical steps in the overarching aim of identifying optimally cost-efficient studies of biomechanical exposure.

## Competing interests

The authors declare that they have no competing interest.

## Authors’ contributions

CT developed the specific research question at hand, contributed to sampling strategy, data collection, and data processing protocols, performed data collection and data processing, drafted the cost model, and drafted the manuscript. SEM conceived of the original study of flight loaders, developed the sampling strategy, contributed to data collection and processing protocols, aided in the development of the cost model, and contributed significantly to the manuscript. JJ contributed to data collection and processing protocols, performed data collection and data processing, and contributed significantly to the manuscript. JW contributed to sampling strategy and data collection protocols, performed data collection and data processing, and contributed significantly to the manuscript. All four authors read and approved the final manuscript.

## Pre-publication history

The pre-publication history for this paper can be accessed here:

http://www.biomedcentral.com/1471-2288/13/124/prepub

## References

[B1] Winkel J Mathiassen SE Assessment of physical work load in epidemiologic studies: concepts, issues and operational considerations Ergonomics 1994 13 6 979 988 10.1080/00140139408963711 8026455

[B2] van der Beek AJ Frings-Dresen MH Assessment of mechanical exposure in ergonomic epidemiology Occup Environ Med 1998 13 5 291 299 10.1136/oem.55.5.291 9764106 PMC1757583

[B3] Rezagholi M Mathiassen SE Cost-efficient design of occupational exposure assessment strategies–a review Ann Occup Hyg 2010 13 8 858 868 10.1093/annhyg/meq072 20926518

[B4] Trask C Mathiassen SE Price versus precision: cost efficiency in trunk posture observation. in Proceeding of the 7th International Scientific Conference on Prevention of Work-Related Musculoskeletal Disorders (PREMUS 2010) CD-rom 2010 Angers, France: University of Angers

[B5] Mathiassen SE Trask C Cost efficiency of adding another subject or another day to an exposure dataset Proceeding of the 7th International Scientific Conference on Prevention of Work-Related Musculoskeletal Disorders (PREMUS 2010) CD-rom 2010 Angers, France (available from University of Angers)

[B6] Mathiassen SE Bolin K Optimizing cost-efficiency in mean exposure assessment - cost functions reconsidered BMC Med Res Methodol 2011 13 76 10.1186/1471-2288-11-76 21600023 PMC3125387

[B7] Mathiassen SE Liv P Wahlström J Cost-efficient measurement strategies for posture observations based on video recordings Appl Ergon 2013 13 609 617 10.1016/j.apergo.2012.12.003 23333111

[B8] Travers EM Cost analysis in the toxicology laboratory Clin Lab Med 1990 13 3 591 623 2253452

[B9] Brown JL Vanable PA Eriksen MD Computer-assisted self-interviews: a cost effectiveness analysis Behav Res Methods 2008 13 1 1 7 10.3758/BRM.40.1.1 18411521 PMC2572260

[B10] Trask C Data collection costs in industrial environments for three occupational posture exposure assessment methods BMC Med Res Methodol 2012 13 89 10.1186/1471-2288-12-89 22738341 PMC3439320

[B11] Rezagholi M Mathiassen SE Liv P Cost efficiency comparison of four video-based techniques for assessing upper arm postures Ergonomics 2012 13 3 350 360 10.1080/00140139.2011.642007 22409172

[B12] Trask C Measuring low back injury risk factors in challenging work environments: an evaluation of cost and feasibility Am J Ind Med 2007 13 9 687 696 10.1002/ajim.20497 17680639

[B13] Mathiassen SE Efficient one-day sampling of mechanical job exposure data--a study based on upper trapezius activity in cleaners and office workers AIHA J (Fairfax, Va) 2003 13 2 196 211 10.1080/15428110308984809 12688844

[B14] Jackson JA Mathiassen SE Dempsey PG Methodological variance associated with normalization of occupational upper trapezius EMG using sub-maximal reference contractions J Electromyogr Kinesiol 2009 13 3 416 427 10.1016/j.jelekin.2007.11.004 18155593

[B15] Liv P Mathiassen SE Svendsen SW Theoretical and empirical efficiency of sampling strategies for estimating upper arm elevation Ann Occup Hyg 2011 13 4 436 449 10.1093/annhyg/meq095 21486917

[B16] Hansson GA Precision of measurements of physical workload during standardised manual handling. Part II: Inclinometry of head, upper back, neck and upper arms J Electromyogr Kinesiol 2006 13 2 125 136 10.1016/j.jelekin.2005.06.009 16102977

[B17] Hansson GA Validity and reliability of triaxial accelerometers for inclinometry in posture analysis Med Biol Eng Comput 2001 13 4 405 413 10.1007/BF02345361 11523728

[B18] Kazmierczak K An integrated analysis of ergonomics and time consumption in Swedish 'craft-type' car disassembly Appl Ergon 2005 13 3 263 273 10.1016/j.apergo.2005.01.010 15854569

[B19] Wahlstrom J Upper arm postures and movements in female hairdressers across four full working days Ann Occup Hyg 2010 13 5 584 594 10.1093/annhyg/meq028 20385661

[B20] Bao S Interrater reliability of posture observations Hum Factors 2009 13 3 292 309 10.1177/0018720809340273 19750793

[B21] Mathiassen SE Burdorf A van der Beek AJ Statistical power and measurement allocation in ergonomic intervention studies assessing upper trapezius EMG amplitude. A case study of assembly work J Electromyogr Kinesiol 2002 13 1 45 57 10.1016/S1050-6411(01)00028-1 11804811

[B22] Galesic M Bosnjak M Effects of Questionnaire Length on Participation and Indicators of Response Quality in a Web Survey Public Opin Q 2009 13 349 360 10.1093/poq/nfp031

[B23] Abernethy AP Improving health care efficiency and quality using tablet personal computers to collect research-quality, patient-reported data Health Serv Res 2008 13 6 1975 1991 10.1111/j.1475-6773.2008.00887.x 18761678 PMC2613994

[B24] Jackson J Punnett L Mathiassen SE Statistical precision of categorical PATH observations of trunk posture Work 2012 13 Supplement 1 5519 5521 22317601 10.3233/WOR-2012-0868-5519

[B25] Kazmierczak K Observer reliability of industrial activity analysis based on video recordings Int J Ind Ergon 2006 13 275 282 10.1016/j.ergon.2005.12.006

[B26] Svendsen SW Work related shoulder disorders: quantitative exposure-response relations with reference to arm posture Occup Environ Med 2004 13 10 844 853 10.1136/oem.2003.010637 15377771 PMC1740658

[B27] Tak S Physical ergonomic hazards in highway tunnel construction: overview from the Construction Occupational Health Program Appl Ergon 2011 13 5 665 671 10.1016/j.apergo.2010.10.001 21112043

[B28] Trask CM EMG estimated mean, peak, and cumulative spinal compression of workers in five heavy industries Int J Ind Ergonom 2010 13 4 448 454 10.1016/j.ergon.2010.02.006

[B29] Burdorf A van der Beek AJ In musculoskeletal epidemiology are we asking the unanswerable in questionnaires on physical load? Scand J Work Environ Health 1999 13 2 81 83 10.5271/sjweh.409 10360462

[B30] Wells R Assessment of physical work load in epidemiologic studies: common measurement metrics for exposure assessment Ergonomics 1997 13 1 51 61 10.1080/001401397188369 8995047

[B31] Spielholz P Comparison of self-report, video observation and direct measurement methods for upper extremity musculoskeletal disorder physical risk factors Ergonomics 2001 13 6 588 613 11373023 10.1080/00140130118050

[B32] Village J Development and evaluation of an observational Back-Exposure Sampling Tool (Back-EST) for work-related back injury risk factors Appl Ergon 2009 13 538 544 10.1016/j.apergo.2008.09.001 18950744

[B33] Fethke NB Variability in muscle activity and wrist motion measurements among workers performing non-cyclic work J Occup Environ Hyg 2012 13 1 25 35 10.1080/15459624.2012.634361 22150404

[B34] Trask CM Optimising sampling strategies: components of low-back EMG variability in five heavy industries Occup Environ Med 2010 13 12 853 860 10.1136/oem.2010.055541 20581418

[B35] Samuels SJ Lemasters GK Carson A Statistical methods for describing occupational exposure measurements Am Ind Hyg Assoc J 1985 13 8 427 433 10.1080/15298668591395111 4050679

[B36] Chen CC Sampling strategies for occupational exposure assessment under generalized linear model Ann Occup Hyg 2009 13 5 509 521 10.1093/annhyg/mep034 19460758

[B37] Hoogendoorn WE Flexion and rotation of the trunk and lifting at work are risk factors for low back pain: results of a prospective cohort study Spine (Phila Pa 1976) 2000 13 23 3087 3092 10.1097/00007632-200012010-00018 11145822

[B38] Ariens GA High quantitative job demands and low coworker support as risk factors for neck pain: results of a prospective cohort study Spine (Phila Pa 1976) 2001 13 17 1896 1901 discussion 1902-3 10.1097/00007632-200109010-00016 11568702

[B39] Burdorf A Jansen JP Predicting the long term course of low back pain and its consequences for sickness absence and associated work disability Occup Environ Med 2006 13 8 522 529 10.1136/oem.2005.019745 16849528 PMC2078120

